# Selenium Improves Yield and Quality in *Prunella vulgaris* by Regulating Antioxidant Defense, Photosynthesis, Growth, Secondary Metabolites, and Gene Expression Under Acid Stress

**DOI:** 10.3390/plants14060920

**Published:** 2025-03-14

**Authors:** Lixia Zhang, Qingshan Chang, Xingli Zhao, Qi Guo, Shuangchen Chen, Qiaoming Zhang, Yinglong He, Sudan Chen, Ke Chen, Ruiguo Ban, Yuhang Hao, Xiaogai Hou

**Affiliations:** 1 College of Agriculture, Henan University of Science and Technology, Luoyang 471000, China; hkdzlx@126.com (L.Z.); hellen1984@haust.edu.cn (X.Z.); guoqi0529@126.com (Q.G.); hyl8282.good@163.com (Y.H.); cc806056239@163.com (K.C.); banrg02118@126.com (R.B.); 2 College of Horticulture and Plant Protection, Henan University of Science and Technology, Luoyang 471000, China; chen_shuangchen@126.com (S.C.); zhangqm1013@163.com (Q.Z.); elims2006@163.com (S.C.); hao22141904@126.com (Y.H.)

**Keywords:** *Prunella vulgaris* L., acid stress, Selenium, photosystem, root morphology, secondary metabolites

## Abstract

*Prunella vulgaris*, an essential component of traditional Chinese medicine, is suitable for growing in soil with a pH value ranging from 6.5 to 7.5. However, it is primarily cultivated in acidic soil regions of China, where its growth is frequently compromised by acidic stress. Selenium (Se) has been recognized for its potential to enhance stress tolerance in plants. However, its role in acid-stress-induced oxidative stress is not clear. In this study, the effects of varying Se concentrations on the growth and quality of *P. vulgaris* under acidic stress were investigated. The results showed that acid stress enhanced antioxidant enzyme activities, non-enzymatic antioxidant substances, and osmolyte content, accompanied by an increase in oxidant production and membrane damage. Furthermore, it decreased the photosynthetic capacity, inhibited root and shoot growth, and diminished the yield of *P. vulgaris*. In contrast, exogenous application of Se, particularly at 5 mg L^−1^, markedly ameliorated these adverse effects. Compared to acid-stressed plants, 5 mg L^−1^ Se treatment enhanced superoxide dismutase, peroxidase, ascorbate peroxidase, and glutathione peroxidase activities by 150.19%, 54.94%, 43.43%, and 45.55%, respectively. Additionally, soluble protein, soluble sugar, and proline contents increased by 11.75%, 23.32%, and 40.39%, respectively. Se application also improved root architecture and alleviated membrane damage by reducing hydrogen peroxide, superoxide anion, malondialdehyde, and electrolyte leakage levels. Furthermore, it significantly enhanced the photosynthetic capacity by elevating pigment levels, the performance of PSI and PSII, electron transfer, and the coordination of PSI and PSII. Consequently, plant growth and spica weight were significantly promoted, with a 12.50% increase in yield. Moreover, Se application upregulated key genes involved in flavonoid and phenolic acid metabolic pathways, leading to elevated levels of total flavonoids, caffeic acid, ferulic acid, rosmarinic acid, and hyperoside by 31.03%, 22.37%, 40.78%, 15.11%, and 20.84%, respectively, compared to acid-stressed plants. In conclusion, exogenous Se effectively alleviated the adverse effects of acid stress by improving the antioxidant system, growth, and photosynthetic capacity under acid stress, thus enhancing the yield and quality of *P. vulgaris*.

## 1. Introduction

Soil acidification can adversely affect plant growth, causing global crop losses to exceed USD 600 million every year [1]. It is principally attributed to a combination of factors, including acid deposition, high-N fertilization, and plant uptake [2]. In recent times, the continuous impact of acidic rainfall and the prevalent utilization of nitrogen fertilizers have markedly accelerated and exacerbated the phenomenon of soil acidification [3]. Globally, the area affected by soil acidification approximates 3.95 billion hm^2^, constituting roughly 30% of the total terrestrial surface area [4]. In China, the acidified soil area is 2.18 million km^2^, which constitutes 22.7% of the entire landmass [5]. Soil acidification alters soil properties, impedes plant development and growth, causes serious membrane lipid peroxidation and serious electrolyte leakage in plants, promotes the accumulation of reactive oxygen species [6], destroys chloroplast structure [7], leads to a significant decline in photosynthetic capacity [8], decreases crop productivity and quality, and in extreme cases, causes plant withering and death [9]. Efforts must be exerted to enhance the acid tolerance of crops, and a thorough examination of their potential acid tolerance mechanisms is imperative to the harvest and quality of crops.

The medicinal use of *Prunella vulgaris* focuses on its spicas, which are teeming with phenolic compounds and flavonoids, specifically caffeic acid, ferulic acid, rosmarinic acid, and hyperoside [10]. These bioactive secondary metabolites are the main active components in *P. vulgaris*, which are widely used as Chinese patent medicines because of their antioxidant, antiviral, anti-leukemia, and anticancer activities, besides other biological activities [11]. They are also an important raw material of herbal tea because of their effects of clearing the liver and purging fire in traditional Chinese medical science [10]. The cultivation of *P. vulgaris* has been expanded in China, especially in Henan, Jiangsu, Hunan, and other provinces, which are also prone to acid rain and soil acidification [12]. Soil acidification, triggered by acid rain and other environmental factors, has become a crucial obstacle impeding the growth and quality of *P. vulgaris*.

Numerous researchers have demonstrated that selenium (Se) can effectively regulate plant growth and development [13,14,15]. As a vital trace element, selenium acts as a cofactor for glutathione peroxidase (GSH-px), a key antioxidant enzyme [16]. Its strong antioxidant capacity can enhance the performance of antioxidant enzymes and non-enzymatic antioxidants, thus eliminating reactive oxygen species (ROS) in plants [17,18]. In *Melissa officinalis*, exogenous selenium-nanoparticles led to a marked improvement in the activity levels of antioxidant enzymes, including superoxide dismutase (SOD), catalase (CAT), and peroxidase (POX), improving their antioxidant capacity and curbing lipid peroxidation in membranes under salt stress [19]. Similarly, Se-treated *Lippia citriodora* plants under salt stress displayed increased antioxidant enzyme efficacies, higher amounts of osmolytes, and reduced electrolyte leakage, malondialdehyde (MDA) production, and hydrogen peroxide (H_2_O_2_) content [20]. Furthermore, selenium application could prompt the synthesis of auxin-like hormones, thereby remodeling the root architecture [21]. In *Isodon rubescens*, selenite (3 μM kg^−1^) promoted the growth of root length [22]. Also, Se promoted chlorophyll (Chl) synthesis, stomatal opening, carbon dioxide (CO_2_) assimilation efficiency, and photosynthesis, contributing to increased crop yields and better quality [23,24,25].

Photosynthesis is the cornerstone of crop yield generation, delivering essential nutrients and energy to sustain the growth and evolution of plants [24]. The process consists of capturing solar energy and facilitating photochemical reactions within the pigment-protein complexes embedded in the thylakoid membrane [26]. Among these complexes, PSI and PSII are central to photosynthesis, converting light energy to chemical energy through light capture, water splitting, and electron transfer [27]. Harmonious functioning of PSI and PSII proved to be an important step in ensuring a smooth photosynthetic cycle [28,29]. Investigations have evidenced that Se has a positive role in enhancing the photosynthetic apparatus [30,31]. Selenium can improve the electron transfer rate and PSII performance, thus improving the photosynthetic capacity of *Perilla frutescens* under cadmium stress [32]. Similarly, selenium application promoted the increase in the F_v_/F_m_ and F_v_/F_o_ parameters related to the PSII performance of *Dracocephalum moldavica* under cadmium stress [33]. However, its effect on the performance of both photosystems under acid stress remains unknown.

Se can also improve nutritional quality and secondary metabolite biosynthesis by modulating sulfur or nitrogen (S/N), hormone, and redox metabolism [34]. Secondary metabolites, notably phenols and flavonoids, contribute significantly to crop growth, quality maintenance, and adaptive responses to abiotic stresses [35]. Se can enhance phenolic acids and flavonoids accumulation by modulating genes involved in phenylpropionic acid metabolism [36]. Selenium application (6 μM kg^−1^) promoted the remarkable accumulation of flavonoids, diterpenoids, and oridonin in *I. rubescens* plants [22]. Similarly, the production of secondary metabolites, notably essential oils, phenolic compounds, and flavonoids, in *L. citriodora* plants was enhanced by selenium under saline stress conditions [20].

In China, the soil selenium content varies widely, from 0.010 to 16.240 mg kg^−1^, with a median of 0.171 mg kg^−1^ and an average of approximately 0.235 mg kg^−1^, which was significantly lower than the global soil Se concentration of 0.40 mg kg^−1^ [37]. Also, the distribution is extremely uneven, and about 72% of areas are deficient in selenium [38]. Although the concentration of selenium in many red soil areas in southern China (mostly acid rain-prone areas) is relatively high (>0.4 mg kg^−1^), it is easily adsorbed by a large number of iron and aluminum oxides in red soil to form iron–aluminum complexes, resulting in a low bioavailability of selenium, with the bioavailability less than 11% [39]. Compared with soil selenium application, foliar selenium spraying, displaying cost-effectiveness, safety, and simplicity, is a superior method, and two common selenium sources, selenite and selenate, are frequently used for this purpose [40,41]. Spraying selenite, in contrast to selenate, offers superior advantages for plant organic selenium production, higher safety for humans and animals, and greater ease of utilization [42,43,44]. Additionally, research findings indicated that most tomato quality attributes exhibited a heightened responsiveness to selenite in contrast to selenate [45], indicating that sodium selenite was a preferable form of selenium fertilizer.

Exogenous selenium application has been proven beneficial in alleviating several abiotic stresses faced by plants, such as salt stress [20,46], drought stress [47], and cadmium stress [48] in plants. However, there is a scarcity of information regarding the impact of selenium in mitigating acidic stress. Therefore, in this study, the influences of acidic stress and Se on the antioxidant system, ROS levels, membrane damage, gas exchange parameters, chlorophyll transient fluorescence characteristics, 820 nm modulation reflection curve, growth, yield traits, and secondary metabolites were examined. Moreover, several key genes involved in phenolic and flavonoid biosynthesis were specifically examined, as they play pivotal roles in secondary metabolite synthesis and are directly linked to the enhancement of pharmaceutically active components like rosmarinic acid in *P. vulgaris*. This research contributes to elucidating the mitigating influence of selenium on acidic stress, providing a reference for further utilizing selenium to regulate the growth and development of medicinal plants under acid stress.

## 2. Results

### 2.1. Antioxidant Enzyme Activity

**Figure 1:**
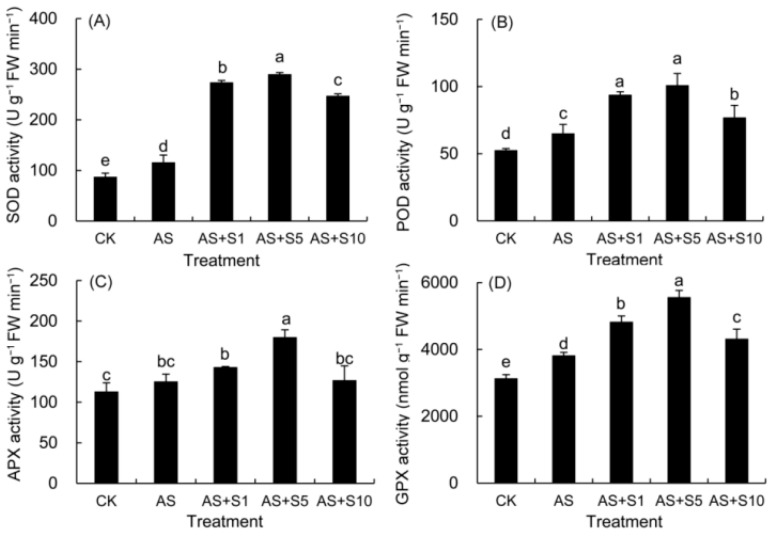
Effects of selenium on activities of SOD (**A**), POD (**B**), APX (**C**) and GPX (**D**) in *P. vulgaris* under acid stress. FW: fresh weight, CK: control. AS: acid stress. AS+S1, AS+S5, and AS+S10: treated with acid stress and 1, 5, and 10 mg L^−1^ Se solution, respectively. After treatment with the selenium spray, the seedlings underwent 15 days of acid stress and were subsequently tested. The data are represented as the mean ± standard deviation. Significant differences are indicated by different lowercase letters above the bars according to a Duncan’s multiple range test (*p* < 0.05).

In this study (Figure 1), the activities of SOD, POD, APX, and GPX enzymes were observed to increase under acidic stress conditions. The activities of these antioxidant enzymes were found to be further bolstered by Se treatments, notably peaking at a selenium concentration of 5 mg L^−1^.

### 2.2. Osmolytes Contents

**Figure 2:**
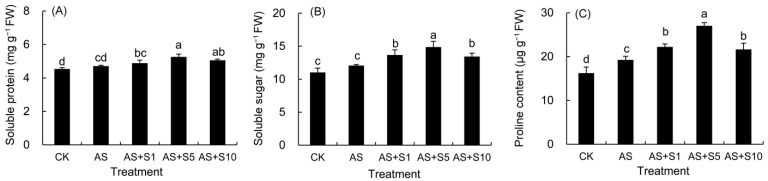
Effects of exogenous selenium on the contents of soluble protein (**A**), soluble sugar (**B**), and proline (**C**) in *P. vulgaris* under acid stress. FW: fresh weight. CK: control. AS: acid stress. AS+S1, AS+S5, and AS+S10: treated with acid stress and 1, 5, and 10 mg L^−1^ Se solution, respectively. After treatment with the selenium spray, the seedlings underwent 15 days of acid stress and were subsequently tested. The data are represented as the mean ± standard deviation. Significant differences are indicated by different lowercase letters above the bars according to a Duncan’s multiple range test (*p* < 0.05).

The contents of soluble protein, soluble sugar, and proline in the leaves of *P. vulgaris* all increased under acid stress (Figure 2). Following selenium application, marked increments in these components under acidic conditions were observed. Notably, 5 mg L^−1^ Se treatment significantly boosted soluble sugar and proline levels compared to 1 and 10 mg L^−1^ Se treatments.

### 2.3. H_2_O_2_, O_2_^−^ and MDA Content and Electrolyte Leakage

**Figure 3:**
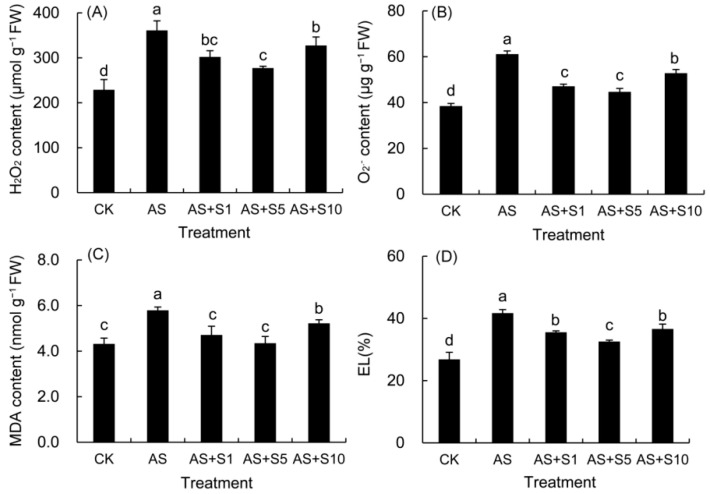
Effects of selenium treatment on the contents of H_2_O_2_ (**A**), O_2_^−^ (**B**), MDA (**C**), and EL (**D**) in *P. vulgaris* under acid stress. FW: fresh weight. CK: control. AS: acid stress. AS+S1, AS+S5, and AS+S10: treated with acid stress and 1, 5, and 10 mg L^−1^ Se solution, respectively. After treatment with the selenium spray, the seedlings underwent 15 days of acid stress and were subsequently tested. The data are represented as the mean ± standard deviation. Significant differences are indicated by different lowercase letters above the bars according to a Duncan’s multiple range test (*p* < 0.05).

In comparison to the control leaves (Figure 3), acid stress significantly elevated the levels of H_2_O_2_, O_2_^−^, and MDA, along with an increase in electrolyte leakage (EL). In contrast, all doses of selenium treatment significantly reduced the values of these four parameters compared to acid stress. In particular, the 5 mg L^−1^ selenium treatment led to the most substantial decline in all four indicators.

### 2.4. Photosynthetic Pigment

**Figure 4:**
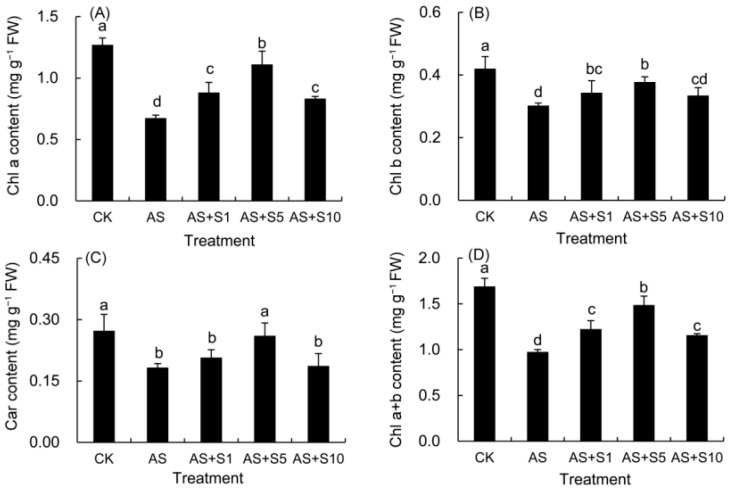
Effects of exogenous selenium on the contents of Chl a (**A**), Chl b (**B**), Car (**C**) and Chl a + b (**D**) in *P. vulgaris* under acid stress. FW: fresh weight. Cha co CK: control. AS: acid stress. AS+S1, AS+S5, and AS+S10: treated with acid stress and 1, 5, and 10 mg L^−1^ Se solution, respectively. After treatment with the selenium spray, the seedlings underwent 15 days of acid stress and were subsequently tested. The data are represented as the mean ± standard deviation. Significant differences are indicated by different lowercase letters above the bars according to a Duncan’s multiple range test (*p* < 0.05).

The contents of Chl a, Chl b, Car, and Chl a + b markedly decreased in the plants cultivated under acid stress compared with those in the control leaves (Figure 4). However, Se treatments effectively promoted the elevation of various pigment components. It is worth noting that 5 mg L^−1^ selenium treatment showed the most significant improvement effect.

### 2.5. Gas Exchange Parameters

**Figure 5:**
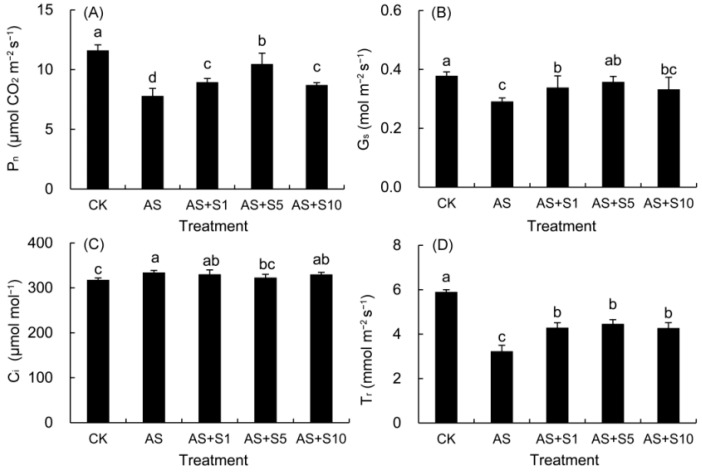
Effects of exogenous selenium on P_n_ (**A**), G_s_ (**B**), C_i_ (**C**), and T_r_ (**D**) in *P. vulgaris* under acid stress. FW: fresh weight. CK: control. AS: acid stress. AS+S1, AS+S5, and AS+S10: treated with acid stress and 1, 5, and 10 mg L^−1^ Se solution, respectively. After treatment with the selenium spray, the seedlings underwent 15 days of acid stress and were subsequently tested. The data are represented as the mean ± standard deviation. Significant differences are indicated by different lowercase letters above the bars according to a Duncan’s multiple range test (*p* < 0.05).

As shown in Figure 5, acid stress induced a marked decrease in the P_n_, G_s_, and T_r_, but a remarkable increase in the C_i_ compared with those in the control leaves. By comparison, the application of selenium to *P. vulgaris* leaves under acid stress led to an augmentation in P_n_, G_s_, and T_r_ and a reduction in C_i_. The most effective treatment was found to be 5 mg L^−1^ selenium.

### 2.6. Root Architecture

In this study, plants subjected to acid stress exhibited a notable decrease in root length, root surface area, root volume, number of root tips, and branch number compared to control plants (Table 1). Conversely, all selenium treatments significantly promoted the root growth of *P. vulgaris* seedlings compared to those under acid stress.

### 2.7. Morphology and Biomass

**Figure 6:**
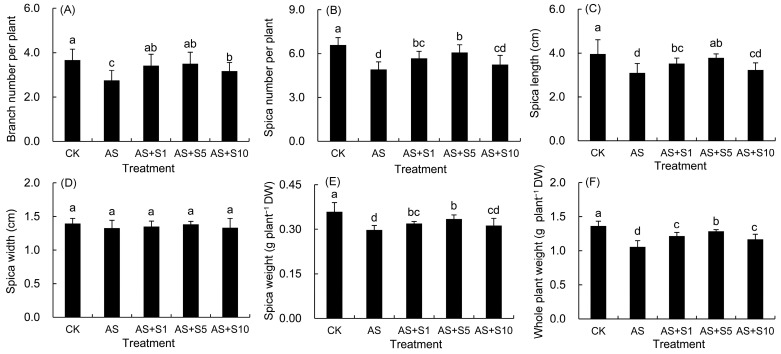
Effects of selenium on morphology and biomass characteristics of P. vulgaris under acid stress (**A**) Branch number per plant; (**B**) Spica number per plant; (**C**) Spica length; (**D**) Spica width; (**E**) Spica weight; (**F**) Whole plant weight. DW: dry weight. CK: control. AS: acid stress. AS+S1, AS+S5, and AS+S10: treated with acid stress and 1, 5, and 10 mg L−1 Se solution, respectively. After treatment with the selenium spray, the seedlings underwent 15 days of acid stress and were subsequently tested. The data are represented as the mean ± standard deviation. Significant differences are indicated by different lowercase letters above the bars according to a Duncan’s multiple range test (*p* < 0.05).

As shown in Figure 6, the branch number per plant, spica number per plant, spica length, spica weight per plant, and total weight per plant of *P. vulgaris* under acid stress significantly decreased than those in the control plants under acid stress. However, compared to exposure to acid stress, selenium could enhance these growth indicators of *P. vulgaris* to varying degrees, with the optimal effect observed under 5 mg L^−1^ Se treatment.

### 2.8. OJIP Curve and 820 nm Modulated Reflection

**Figure 7:**
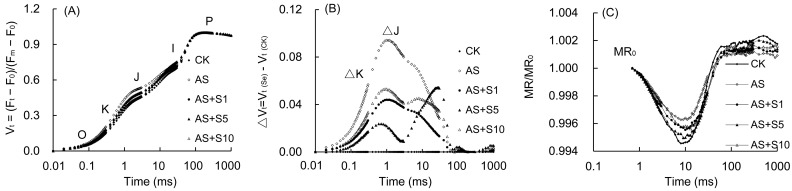
The V_t_ curves (**A**), △V_t_ curves (**B**), and 820 nm modulated reflection curve (**C**) of *P. vulgaris* under different Se treatments. V_t_ = (F_t_ − F_0_)/(F_m_ − F_0_): the relative variable fluorescence (Vt) at any time; △V_t_ = V_t (Se)_ − V_t (CK)_; O, K, J, I, and P represent different phases in the OJIP curve. MR/MR_0_: the 820 nm modulated reflection curve; MR is the modulated reflection at different time points; MR_0_ is the MR value of far-red light irradiated at 0.7 ms. After treatment with the selenium spray, the seedlings underwent 15 days of acid stress and were subsequently tested. CK: control. AS: acid stress. AS+S1, AS+S5, and AS+S10: treated with acid stress and 1, 5, and 10 mg L^−1^ Se solution, respectively.

The OJIP curve (V_t_ curves) was derived by means of the process of normalizing the step from O to P (Figure 7A). In comparison with the control plants, the shape of the V_t_ curve underwent notable alterations under acid stress, particularly during the K and J phases. When subjected to acid stress, the ΔV_t_ curve (Figure 7B) exhibited a positive shift (ΔK, ΔJ > 0) in the K and J points at 300 μs and 2 ms, with the J point showing a more pronounced increase than the K point when contrasted with the control plants. However, Se greatly declined the K and J phases in the OJIP curves relative to acid stress. The analysis of the ΔV_t_ curve revealed that the K and J phases after selenium treatment were significantly reduced compared to those under acid stress. The MR/MR_0_ of the *P. vulgaris* 820 nm reflection curve underwent a decrease from 0.7 ms (Figure 7B) to its nadir between 3 and 30 ms, followed by a steady increase, culminating in a maximum at approximately 300 ms. The minimum MR/MR_0_ ratio, when subjected to acid stress, was observed to be markedly elevated compared with the control. After Se treatment, the minimum value of MR/MR_0_ decreased significantly compared with that after acid stress treatment.

### 2.9. JIP Parameters

**Figure 8:**
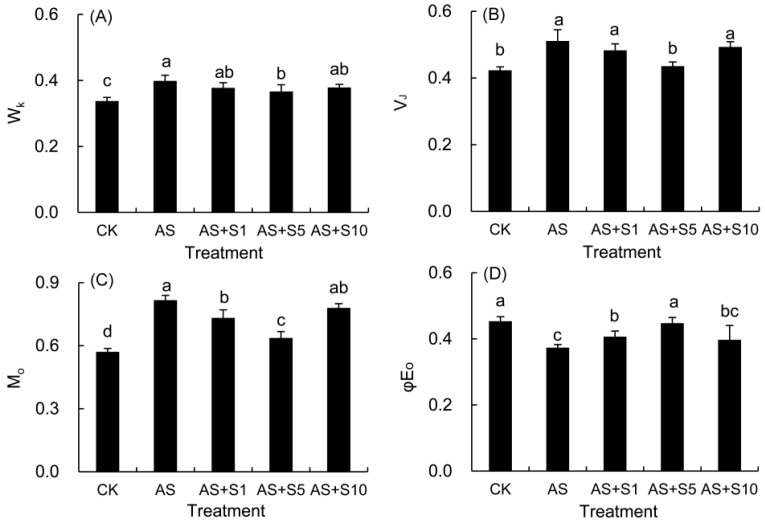
Effects of selenium on W_k_ (**A**), V_J_ (**B**), M_0_ (**C**), and φE_0_ (**D**) in *P. vulgaris* under acid stress. CK: control. AS: acid stress. AS+S1, AS+S5, and AS+S10: treated with acid stress and 1, 5, and 10 mg L^−1^ Se solution, respectively. After treatment with the selenium spray, the seedlings underwent 15 days of acid stress and were subsequently tested. The data are represented as the mean ± standard deviation. Significant differences are indicated by different lowercase letters above the bars according to a Duncan’s multiple range test (*p* < 0.05).

The normalized fluorescence of the K (W_K_) and the J phases (V_J_) was quantitatively assessed by analyzing the variations in these specific phases within the OJIP curve (Figure 8A,B). Subjected to acid stress, the W_K_, V_J_, and M_0_ measurements rose by 18.16%, 20.83%, and 43.09%, respectively, in comparison to the control plants. After Se application, the three parameters decreased to varying extents, with the most significant reduction observed in the 5 mg L^−1^ selenium treatment. As opposed to the control, the φE_0_ value decreased significantly in response to acid stress, but it increased significantly when treated with 1 and 5 mg L^−1^ selenium.

When compared with the control, acid stress led to a substantial elevation in ABS/RC, DI_0_/RC, TR_0_/RC, and φD_0_ parameters (Table 2). The values of the aforementioned four parameters decreased after Se treatment; the maximum decrease was observed under 5 mg L^−1^ Se treatment. Despite a non-significant increase in ET_0_/RC under acid stress with selenium supplementation, there was a marked decline in ABS/CS_m_, TR_0_/CS_m_, ET_0_/CS_m_, and reaction center density (RC/CS_m_) per cross section under acidic conditions, when compared to the control. Selenium application resulted in an elevation of these four parameter values and a reduction in DI_0_/CS_m_ under acid stress, with the most pronounced effect under 5 mg L^−1^ treatment.

### 2.10. The Function and Coordination of PSII and PSI

**Figure 9:**
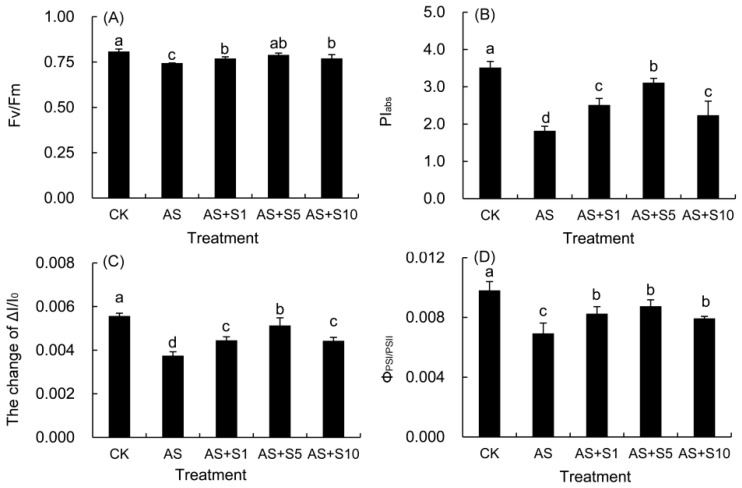
The function and coordination of PSII and PSI in *P. vulgaris* treated with Se under acid stress. F_v_/F_m_ (**A**); PI_abs_ (**B**); ΔI/I_0_ (**C**); and Φ_PSI/PSII_ (**D**). CK: control. AS: acid stress. AS+S1, AS+S5, and AS+S10: treated with acid stress and 1, 5, and 10 mg L^−1^ Se solution, respectively. After treatment with the selenium spray, the seedlings underwent 15 days of acid stress and were subsequently tested. The data are represented as the mean ± standard deviation. Significant differences are indicated by different lowercase letters above the bars according to a Duncan’s multiple range test (*p* < 0.05).

The values of F_v_/F_m_ and PI_abs_ in *P. vulgaris* exhibited a marked decline under acid stress compared with the control plants (Figure 9A,B). Nevertheless, the addition of exogenous selenium markedly raised the levels of these two parameters. The F_v_/F_m_ and PI_abs_ values under all Se treatments were significantly elevated compared to those under acid stress. Notably, the peak values were attained under the 5 mg L^−1^ Se treatment.

The ΔI/I_0_ and Φ_PSI/PSII_ under acid stress significantly decreased than those in the control plants (Figure 9C,D), but considerably increased after Se treatments. At a concentration of 5 mg L^−1^ of selenium, the two parameters increased the most.

### 2.11. The Contents of Secondary Metabolites

Acid-stressed plants exhibited elevated levels of total flavonoids, caffeic acid, ferulic acid, rosmarinic acid, and hyperoside compared to control plants (Table 3). Upon selenium treatment, the contents of these active components were significantly augmented compared with those under acid stress. Additionally, 5 mg L^−1^ Se treatment yielded the optimal results in increasing the levels of total flavonoids (31.03%), caffeic acid (22.37%), ferulic acid (40.78%), rosmarinic acid (15.11%), and hyperoside (20.84%) compared to acid stress treatment. In both 5 and 10 mg L^−1^ selenium treatments, the levels of total flavonoids, caffeic acid, rosmarinic acid, and hyperoside were comparable; however, the content of ferulic acid in 10 mg L^−1^ treatment was notably lower.

### 2.12. Expression Level of Key Genes

**Figure 10:**
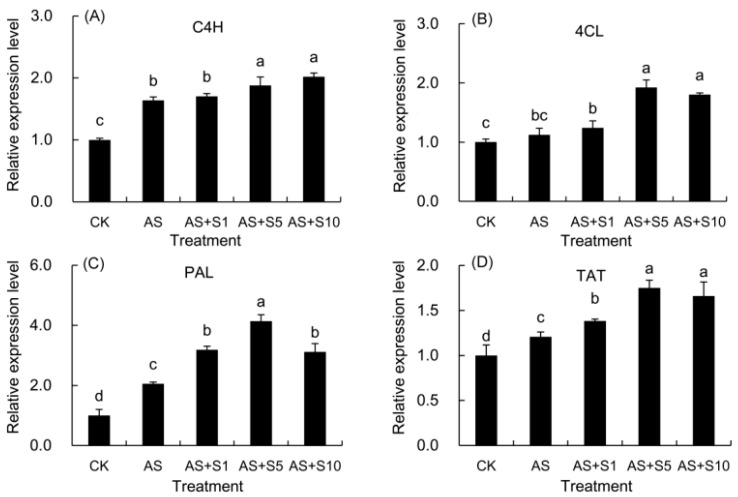
The transcript abundance of rosmarinic acid biosynthesis genes of plants treated with Se. (**A**) C4H coumarate 4-hydroxylase; (**B**) 4CL 4-coumaroyl CoA ligase; (**C**) PAL phenylalanine ammonia-lyase; (**D**) TAT tyrosine aminotransferase. CK: control. AS: acid stress. AS+S1, AS+S5, and AS+S10: treated with acid stress and 1, 5, and 10 mg L^−1^ Se solution, respectively. After treatment with the selenium spray, the seedlings underwent 15 days of acid stress and were subsequently tested. The data are represented as the mean ± standard deviation. Significant differences are indicated by different lowercase letters above the bars according to a Duncan’s multiple range test (*p* < 0.05).

Acid stress upregulated 4-coumaroyl CoA ligase (*4CL*) and markedly enhanced coumarate 4-hydroxylase (*C4H*)*,* phenylalanine ammonia-lyase (*PAL*), and tyrosine aminotransferase (*TAT*) gene expression compared with the control (Figure 10). Upon application of various selenium concentrations, the *C4H* gene exhibited a steadily rising trend in expression, significantly surpassing acid stress except at 1 mg L^−1^. For the *4CL* gene, its expression was enhanced above acid stress levels at 1 mg L^−1^ selenium treatment, with even more pronounced elevations observed at 5 and 10 mg L^−1^ selenium treatments. Interestingly, the *PAL* and *TAT* genes displayed a similar trend, where their expression surpassed that of the acid stress control significantly in all selenium treatments. Furthermore, as the selenium concentration increased, their expression initially rose and then declined. It is noteworthy that the 5 mg L^−1^ selenium treatment was the most effective, outperforming all other tested concentrations in terms of its influence on the expression patterns of the *4CL*, *PAL*, and *TAT* genes.

## 3. Discussion

Acid stress induced oxidative damage by disrupting ROS metabolic balance, while exogenous selenium maintained cellular redox homeostasis through synergistic effects of enzymatic and non-enzymatic antioxidant systems. The balance between ROS production and clearance in plants is dynamic, but stress conditions, such as acid stress, often lead to an excessive accumulation of ROS, thereby disrupting this equilibrium [17]. In the antioxidant system, O_2_^−^ is first disproportionated by SOD into H_2_O_2_ and O_2_, and H_2_O_2_ is further metabolized into O_2_ and H_2_O by enzymes such as POD, APX, and GPX [49], thereby preventing ROS from causing more damage to plant cells. In this study, we observed that acid stress significantly increased the levels of H_2_O_2_ and O_2_^−^ in *P. vulgaris* (Figure 3) by 57.61% and 58.96%, respectively, accompanied by a 55.59% rise in electrolyte leakage (EL) and a 34.04% increase in malondialdehyde (MDA) content, indicating severe membrane lipid damage. These results suggest that acid stress disrupted the ROS metabolic balance, exacerbating oxidative damage in plant cells. Selenium, as a micronutrient, has been shown to regulate the plant antioxidant system through multiple mechanisms. Firstly, Se can be metabolized in plants to form Se-containing organic derivatives, such as selenocysteine and selenomethionine, which directly participate in scavenging ROS and maintaining cell homeostasis [50]. Secondly, selenium can facilitate the spontaneous conversion of O_2_^−^ into H_2_O_2_ without SOD enzyme catalysis [17,51]. Additionally, selenium can serve as a component within enzyme complexes, activating enzyme function, including selenium-dependent GPX [52,53]. Selenium can not only upregulate the expression of genes encoding antioxidant enzymes such as SOD, POD, APX, and CAT, enhancing their activities [14,54] and indirectly increasing the content of non-enzymatic antioxidants [51], but also downregulate the expression of genes involved in reactive oxygen species (ROS) generation, such as NADPH oxidase (*BnaNOXs*) and glycolate oxidase (*BnaGLO*), thus reducing ROS levels [55]. In our study, Se supplementation (5 mg L^−1^) significantly enhanced the activities of antioxidant enzymes (Figure 1), with SOD activity increasing by 150.19%, POD by 54.94%, APX by 43.43%, and GPX by 45.55%. Concurrently, ROS levels (Figure 3) were substantially reduced, with H_2_O_2_ decreasing by 23.18% and O_2_•^−^ by 26.89%, while membrane damage indicators such as EL and MDA declined by 21.96% and 24.88%, respectively. These results strongly suggest that selenium effectively strengthened the antioxidant defenses in the leaves, scavenging ROS and maintaining cellular stability and membrane integrity. Comparable findings were also reported in previous investigations focusing on selenium-enhanced *M. officinalis* [19] plants exposed to cadmium stress [48] and selenium-treated *D. moldavica* [33] plants subjected to salt stress [56].

In the process of plants responding to adverse stress, osmolytes play a crucial role. In our experiments, 5 mg L^−1^ Se treatment elevated soluble protein content (Figure 2) by 11.75%, with more pronounced increases in soluble sugars (23.32%) and proline (40.39%) compared to the acid-stressed treatment. Pretreatment with selenium resulted in a surge in *OsNAC5* gene expression in rice, playing a part in osmolyte synthesis and thus encouraging the accumulation of proline and soluble sugars [17,57]. Selenium also improved the efficiency of sucrose synthase in the starch–sucrose metabolic pathway, upregulated the *P5CS* gene (Δ1-pyrroline-5-carboxylate synthase) expression in proline biosynthesis, and intensified the enzymatic activity related to nitrogen metabolism in amino acid metabolism [58], which might explain the rise in the above osmolytes of *P. vulgaris*. Furthermore, soluble sugars and proline, acting as non-enzymatic antioxidants, interacted with ROS by donating or accepting electrons, thereby eliminating the highly reactive electron pairs of ROS and reducing their toxicity [15]. The introduction of selenium in the study had a pronounced effect on enhancing the three indicators under acidic conditions, teaming up with antioxidant enzymes to neutralize ROS, minimize harm to the plasma membrane, adjust cellular osmotic pressure, and uphold cellular structural stability [17]. In previous studies, similar enhancements were also observed in *D. moldavica* [33] exposed to selenium and cadmium toxicity stress [47] and in *L. citriodora* given selenium under saline stress [20].

Although acid stress can significantly reduce the photosynthetic pigment content and photosynthetic efficiency of plants, the application of selenium can effectively reverse this trend. Researchers have found that acid-induced stress in plants leads to an overproduction of ROS, which inflicted severe damage on the chloroplast membrane, causing its disintegration and accelerating the breakdown of chlorophyll [59]. Consequently, acid stress treatment demonstrated a significant 42.28% decrease in total chlorophyll content (Figure 4), which was consistent with the significant decline in P_n_ (Figure 5) and PI_abs_ (Figure 9) of *P. vulgaris*. Previous research in *P. vulgaris* [8] and *Salvia meiliensis* [60] likewise documented comparable results under acid stress. In addition to capturing and relaying light energy, carotenoids play a role in scavenging ROS, thereby protecting chlorophyll from photooxidative damage through the xanthophyll cycle [61]. The obvious decline in carotenoid content (33.06%) due to acid stress contributed to the photooxidative damage of chlorophyll of *P. vulgaris* (Figure 4). Yet, investigations have revealed that selenium application can facilitate the intake of nitrogen and magnesium, essential structural elements of chlorophyll [62], and minimized the damage to chloroplasts under abiotic stress conditions [63]. In addition, Se application effectively suppressed the degradation of chlorophyll by downregulating the expression of chlorophyllase (*CHLASE*) [18]. Simultaneously, it activated genes responsible for chlorophyll synthesis, such as protoporphyrinogen oxidase (*PPO*) and porphobilinogen deaminase (*PBGD*), thus promoting the increase in chlorophyll [24]. Se application also enhanced the Car content by up-regulating carotenoid biosynthesis genes, such as farnesyl pyrophosphate synthase 1 (*FPPS1*), phytoene synthase (*PSY2*), and β-carotenoid hydroxylase (*BCH2*) [64]. Therefore, selenium supplementation (5 mg L^−1^) significantly boosted the total chlorophyll (52.51%) and carotenoid content (42.63%) in *P. vulgaris* grown under acidic environments (Figure 4). Previous studies have shown that selenium treatment in *L. citriodora* [20] and (*Plectranthus scutellarioides*) [65] plants similarly resulted in increased pigment levels under salt stress, which is consistent with this study. As stated by Yang et al. [66], each photosynthetic reaction center comprises approximately 300 chlorophyll molecules. Hence, the marked rise in total chlorophyll (Figure 4) content (52.51%) greatly contributed to the increase in the reaction center density (22.32%) per cross section (Table 2) of selenium-treated plants under acid stress.

The dynamic change in photosynthetic gas exchange parameters reveals the complex regulation mechanism of acid stress on photosynthetic carbon assimilation, and the difference in treatment time is a key variable leading to the disagreement of research conclusions. In this study, the gas exchange parameters showed that acid stress induced a marked decline in P_n_ (32.83%) and G_s_ (23.05%), but a significant increase in C_i_ (5.13%), which indicated that the limiting factor for photosynthetic rate was non-stomatal factors rather than insufficient supply of CO_2_ (Figure 5). Nevertheless, the findings from this research did not align with prior investigations in *P. vulgaris* under acid rain stress (pH 4.0); this inconsistency might be attributed to the shorter treatment duration (7 days) of acid rain stress in previous studies, as such a timeframe was insufficient to inflict substantial damage on the photosynthetic apparatus, thereby not triggering an escalation in the C_i_ values [8]. Compared with the previous short-term acid stress (pH 4.0) study for 7 days, the 15-day treatment duration in this study allowed the damage caused by acid stress to the photosynthetic apparatus to fully accumulate and manifest. During the short-term 7-day stress, plants may only initiate preliminary stress defense and do not cause substantial damage to the photosynthetic apparatus that affects carbon dioxide assimilation, so there is no significant increase in Ci value [8]. Under 15-day acid stress, the net photosynthetic rate (P_n_) of *P. vulgaris* decreased (Figure 5), which was due to the accumulation of intracellular hydrogen ions and toxic ions (SO_4_^2−^, NO_3_^−^, Al^3+^, Pb^2+^), resulting in the impairment of the OEC function on the donor side of PSII (Figure 8) and the blockage of electron transfer on the acceptor side, a sharp decline in the density of active reaction centers per cross section (Table 2) accompanied by a decrease in electron transfer capacity (Figure 8). These changes, accompanied by a significant decrease in chlorophyll content, the accumulation of ROS, and severe damage to the cell membrane (Figure 3), jointly led to the non-stomatal-limitation-dominated inhibition of Pn and the abnormal increase in Ci (Figure 5). In addition, the significant decrease in F_v_/F_m_ and PI_abs_ (Figure 9) further confirmed the substantial damage to the photosynthetic apparatus.

In response to acid-stress-induced photosynthetic damage, exogenous selenium achieves functional remodeling of photosynthetic apparatus through multi-level synergistic mechanisms. In response to acid stress-induced photosynthetic damage in our research, exogenous selenium achieves functional remodeling of photosynthetic apparatus through multi-level synergistic mechanisms. The decline in P_n_ (32.83%) within *P. vulgaris* (Figure 5) when subjected to acid stress could be due to the buildup of intracellular hydrogen ions and toxic ions (e.g., SO_4_^2−^, NO_3_^−^, Al^3+^, Pb^2+^), which disrupt the structure of chloroplasts and the operation of photosystem II, leading to a drop in chlorophyll levels, interference with electron transport, and inadequate production of ATP and NADPH [67,68]. Selenium application mitigated the harm inflicted by acid stress in *P. vulgaris* seedlings, increased G_s_ (22.80%) and T_r_ (38.09%), decreased C_i_ (3.41%), and promoted P_n_ (11.71%) in *P. vulgaris* seedlings (Figure 5). The improved photosynthetic capacity was attributed to selenium supplementation, resulting in heightened antioxidant enzyme activity and thylakoid membrane protection [69], along with a rise in chlorophyll levels [63]. The improvement in RC performance observed with Se were due to the increased expression of the D1 protein from the *psbA* gene, the CP47 protein from the *psbB* gene, and the CP43 protein from the *psbC* gene, all of which are constituents of the PSII reaction center complex [70]. By promoting the expression of light-harvesting pigment proteins, Se has demonstrated its capacity to shield photosynthetic pigments, such as light-harvesting pigment complex I Chl a/b binding protein 2 and light-harvesting pigment complex II Chl a/b binding protein 1 [71,72]. Furthermore, it promoted the assimilation of CO_2_ by enhancing the accumulation of NADPH and ATP [24], ultimately resulting in an upregulation of P_n_ (11.71%) of *P. vulgaris* treated with selenium (5 mg L^−1^) under acid stress. Similarly, previous studies showed that selenium promoted the photosynthesis of *P. frutescens* under Cd stress [32].

Researchers have found that acid stress significantly impacts root morphology and function, while selenium application demonstrates a notable ameliorative effect [13,22]. This research revealed that acid stress markedly suppressed the total root length (54.89%), root surface area (61.62%), root volume (69.02%), root tip number (57.89%), and branch number (63.01%) of *P. vulgaris* (Table 1). The activity of various plant enzymes, notably those pertaining to photosynthesis and respiration, is largely regulated by the levels of potassium (K) and magnesium (Mg) in the plant, with absorption of these elements being affected by the plasma membrane H^+^-ATPase [73]. Nevertheless, exposure to acidic stress resulted in diminished activity of the plasma membrane H^+^-ATPase, which in turn led to stunted root growth [74]. Therefore, acid stress not only slowed root growth but also reduced the intake of vital nutrients like K and Mg by the root system, resulting in inadequate accumulation of materials needed for the biomass of *P. vulgaris*. In contrast, selenium promoted the expansion of both the main and secondary roots by increasing the levels of auxin [13]. The use of selenium enhanced the activity of genes linked to sucrose synthase enzymes, which play a role in the starch–sucrose synthesis pathway, including enzymes like sucrose phosphate synthase, sucrose phosphatase, and sucrose synthase, thus facilitating the supply of energy and carbon skeletons vital for root development [58]. Furthermore, the introduction of selenium boosted auxin concentrations by enhancing the transcription of the auxin synthesis gene (*YUCCA*) and the expression of auxin transport proteins (*PIN*) [13,75]. So, 5 mg L^−1^ Se treatment significantly increased the total root length (102.69%), root surface area (87.30%), root volume (76.77%), root tip number (193.67%), and branch number (145.00%) in *P. vulgaris* facing acid stress (Table 1). Previous studies showed that selenium application could promote the root growth of *I. rubescens* [22], which proved the positive effect of selenium on root development.

Acid stress induced severe membrane lipid peroxidation and biomass reduction in *P. vulgaris*, while selenium application not only alleviated oxidative damage but also enhanced metabolic efficiency and photosynthetic capacity. This study showed that acid stress caused a significant decrease (Figure 3) in EL by 55.59% and MDA by 34.04%, which caused severe cell membrane lipid peroxidation and electrolyte leakage of *P. vulgaris*. Additionally, it reduced the total root length (54.89%) and P_n_ (32.83%) of *P. vulgaris* plants (Table 1). As a consequence, the growth of *P. vulgaris* was substantially reduced by acid stress, resulting in a significant 22.64% decrease in total weight per plant (Figure 6) under acidic conditions. Conversely, Se application enhanced antioxidant capacity and chlorophyll content under acid stress, reduced ROS accumulation, protected the membrane systems essential for photosynthesis and respiration, and enhanced the organization and efficiency of the photosynthetic organs [30,63]. Selenium application further enhanced the activity of genes related to starch and sucrose metabolic processes, improving root absorption and the photosynthetic capacity of leaves, and ultimately facilitating ample energy substrates for carbohydrate synthesis and the build-up of dry matter [76,77]. In this study, the significant increases in the weight of the spica weight per plant (12.50%) and total weight per plant (21.69%) of *P. vulgaris* observed in 5 mg L^−1^ Se treatment (Figure 6) further confirmed the viewpoints of previous researchers. Previous studies have shown that selenium application substantially increased the dry weight of *P. frutescens* [32] seedlings under Cd stress. The results of this study were in agreement with earlier investigations.

Acid stress triggers ROS burst through dual inhibition of electron transfer on both the donor and acceptor sides of PSII, while exogenous selenium alleviates oxidative damage by repairing photosystem function and stabilizing membrane structure. W_k_ represents the inhibition of the oxygen-evolving complex (OEC) of the PSII donor side by stress, a crucial component in the photooxidation of water during photosynthesis. Notably, the application of acid stress to *P. vulgaris* resulted in a notable increase in the W_k_ value (18.16%), indicating a substantial impairment of OEC and significant hindrance to electron transfer within the donor side (Figure 8). The rise in V_J_ (20.83%) and the fall in φE_o_ (17.62%) in this study suggested that there was considerable harm to the electron transfer dynamics involving quinones [78]. M_0_, a measure of the maximum rate of PSII reaction center closure, exhibited a notable increase (43.06%) in this study (Figure 8), suggesting that acid stress significantly inhibited the receptor side of PSII, thereby reducing the efficiency/likelihood of electron transfer from PSII to the PSI receptor side [31]. The greater increase in V_J_ compared to W_K_ indicated higher damage on the receptor side than that on the donor side under acid stress. The serious obstruction of electron transfer resulted in electron leakage, which subsequently reacted with O_2_ within the cells to form ROS, the majority of these ROS were singlet ROS, leading to membrane lipid peroxidation [79]. Therefore, the inhibition of electron transfer mechanism by acid stress in this study led to a significant increase in H_2_O_2_ (57.61%) and O_2_^−^ (58.96%), which further destroyed the membrane structure and caused electrolyte leakage. In this study, W_K_ (8.05%), V_J_ (14.79%), and M_0_ (22.03%) decreased and φE_0_ (19.80%) increased in 5 mg L^−1^ Se treatment, indicating that Se application enhanced OEC activity and enhanced PSII electron transport, effectively reducing electron leakage and ROS accumulation, and preserving the stability of the cell membrane structure under acid stress.

Acid stress significantly reduced PSII photochemical efficiency through the inhibition of light energy absorption, capture, and transfer, while plants maintained electron transport chain function by redistributing excitation energy dissipation. As this study showed, the notable decline in ABS/CS_m_ (9.86%), TR_0_/CS_m_ (13.49%), and ET_0_/CS_m_ (25.69%) indicated that acid stress decreased the absorption, capture, and electron transfer ability of light energy (Table 2). This stress also resulted in a decrease in RC/CS_m_ (26.09%), encouraging a rise in efficiency within the remaining active reactive centers to make up for the loss. Therefore, the efficiency of light absorption (19.88%), capture (16.57%), and heat dissipation (32.02%) in an individual reaction center was markedly enhanced under acid stress (Table 2). However, ET_0_/RC (0.94%), ET_0_/CS_m_ (25.69%), and φE_0_ (17.62%) decreased, while DI_0_/RC (32.02%), DI_0_/CS_m_ (4.35%), and φD_0_ (33.22%) increased under acid stress (Table 2). The findings implied that a lower level of energy was being allocated for electron transfer, and the additional excitation energy in the PSII antenna was being dissipated as heat through non-photochemical quenching, thereby preserving the integrity of the electron transport chain and lessening the harmful impact of photoinhibition on the leaf tissue [80].

Acid stress induced photosystem II dysfunction through dual inhibition of electron transfer on both the donor and acceptor sides, while exogenous selenium enhanced PSII photochemical efficiency by repairing OEC activity and optimizing energy allocation. In this study, the absorption (9.31%), capture (6.59%), and heat dissipation (20.60%) per reaction center demonstrated a marked decline, whereas ET_0_/RC (8.34%) under 5 mg L^−1^ selenium treatment (Table 2) was elevated compared to that under acid stress. Moreover, Se treatment significantly augmented RC/CS_m_ (22.32%) under acid stress. Simultaneously, the energy absorption (7.62%), capture (11.62%), and electron transfer (28.84%) per cross section were significantly augmented, indicating that Se substantially reduced the energy charge per reaction center, thereby minimizing the occurrence of photoinhibition. Specifically, DI_0_/CS_m_ (5.40%%) and φD_0_ (17.62%) decreased, while ET_0_/CS_m_ (28.84%) and RC/CS_m_ (22.32%) significantly increased in 5 mg L^−1^ Se treatment (Table 2), suggesting that Se treatment reduced the excess excitation energy for heat dissipation, thereby enhancing electron transfer efficiency and PSII performance [28,66].

F_v_/F_m_ and PI_abs_, as key indicators of photosystem II function, reveal the extent of photoinhibition damage under acid stress and the repair effect of exogenous selenium through their coordinated changes. The F_v_/F_m_ index, a reliable indicator of photoinhibition, showed a notable decline under acid stress in this study. This decrease suggested severe damage to the PSII complex in *P. vulgaris* under acid stress. Additionally, the photochemical performance index (PI_abs_) provides a comprehensive evaluation of PSII activity [81]. The notable reduction in both F_v_/F_m_ (7.88%) and PI_abs_ (48.26%) under acidic stress indicated significant photoinhibition of PSII and a considerable decrease in photosynthetic efficiency (Figure 9). Following the application of Se, a marked rise in the values of F_v_/F_m_ (6.04%) and PI_abs_ (70.92%) was detected with 5 mg L^−1^ Se treatment, indicating that the exogenous Se effectively mitigated the damage caused by photoinhibition to *P. vulgaris*, contributing to an improvement in its PSII performance under acid stress.

Although acid stress seriously damaged the functions of photosystem II and PSI, the application of selenium not only restored PSI activity, but also significantly improved photosynthetic performance by coordinating electron transfer between the two photosystems. The modulated reflectance curve at 820 nm is utilized to evaluate the redox activity of PSI, while the MR/MR_0_ ratio indicates the capacity of the PSI reaction center to reduce the final electron acceptors [66]. The ΔI/I_0_ index serves as a key metric for assessing PSI performance, reflecting its maximum redox potential. Moreover, Φ_PSI/PSII_ (ΔI/I_0_/ψ_0_) serves as an assessment of the synchronization between PSII and PSI [28]. In this study, the marked decline in ΔI/I_0_ (32.66%) under acid stress suggested substantial damage to PSI functionality, impeding the flow of electrons from PSII to PSI [82]. As previously mentioned, electron transport was also severely hindered in PSII, leading to a notable decline in the performance of both photosystems. When subjected to acid stress, a notable surge in the minimum MR/MR_0_ was observed, accompanied by a pronounced decline in ΔI/I_0_ (32.66%) and Φ_PSI/PSII_ (29.40%), revealing substantial damage to PSI functionality and the disruption of harmony between PSII and PSI. However, the elevation in these two indexes indicated that Se application significantly boosted PSI activity, enhanced the ability of electron transfer from PSII to PSI, and better synchronized the two photosystems, consequently enhancing photosynthetic performance under acid stress. Comparable findings have been reported in *P. frutescens* [32]. The improvement of chlorophyll fluorescence parameters might be attributed to the augmentation of chlorophyll content and the improvement of chloroplast function by Se [83].

Acid stress promoted the accumulation of medicinal active components by activating key genes in the phenylpropanoid pathway, while exogenous selenium further enhanced secondary metabolite synthesis through coordinated regulation of phenylalanine and tyrosine metabolic pathways. Hyperoside is a flavonoid with antioxidation, apoptosis inhibition, and anti-inflammation functions and plays a potential role in treating Alzheimer’s disease [84]. Rosmarinic acid, identified as a key phenolic compound, is appointed as a quality marker for *P. vulgaris* in the Chinese pharmacopeia [85]. Additionally, ferulic acid and caffeic acid are also phenolic acids, with antioxidation, anti-inflammatory, and anticancer effects [10,28]. Under acid stress, the level of bioactive compounds in *P. vulgaris* increased significantly, which can be attributed to the significant changes in gene expression of *P. vulgaris* caused by acid stress of our study, with the expression levels of the genes *PvC4H*, *Pv4CL*, *PvPAL*, and *PvTAT* increasing by 63.73%, 12.32%, 105.65%, and 20.74%, respectively (Figure 10). When treated with 5 mg L^−1^ selenium, the transcriptional activity of these genes showed a synergistic enhancement effect. Compared with acid stress alone, the expression levels of *PvC4H*, *Pv4CL*, *PvPAL*, and *PvTAT* in the selenium-treated group increased by an additional 14.67%, 71.12%, 101.43%, and 44.97%, respectively (Figure 10). These changes in gene expression were directly reflected in the accumulation of metabolites. In particular, under selenium treatment, the contents of total flavonoids (31.03%), caffeic acid (22.37%), ferulic acid (40.78%), rosmarinic acid (15.11%), and hyperoside (20.84%) all increased significantly (Table 3). Flavonoids are synthesized by phenylpropanoid metabolic biosynthesis pathway [36]. The biosynthesis of phenolic acids depends on the phenylalanine and tyrosine pathways. Furthermore, four genes (*PvC4H*, *Pv4CL*, and *PvPAL*) in the phenylalanine pathway and tyrosine aminotransferase (*PvTAT*) in the tyrosine pathway were up-regulated in the acid stress and selenium treatments, which promoted the increase in rosmarinic acid, ferulic acid, and caffeic acid content under acid stress. The relative quantitative expression of these key genes was in alignment with the quantities of medicinal components present in *P. vulgaris*. Similarly, selenium treatment led to the up-regulation of *PAL* involved in phenylalanine biosynthesis and promoted the accumulation of rosmarinic acid in *M. officinalis* under salt stress [19]. Prior investigations evidenced that selenium supplementation could lead to improved synthesis and metabolism of flavonoids and phenols, supporting the conclusions of our study. Selenium application fostered the accumulation of phenolic compounds and flavonoids, which improved their ROS-scavenging activity, resulting in a reduction in membrane lipid peroxidation and electrolyte leakage in *P. vulgaris*. Similarly, the application of Se has been noted to efficiently raise the content of essential oils, total phenolic compounds, and flavonoids in *L. citriodora* under salt stress conditions [20].

## 4. Materials and Methods

### 4.1. Plant Materials

*P. vulgaris* seeds underwent disinfection by immersion in a 10% sodium hypochlorite solution and were rinsed carefully with running tap water, sprinkled evenly into the watered experimental field, and then covered with 0.5 cm of dry fine soil in October 2022. The seedlings were watered promptly to ensure normal growth. In April 2023, the seedlings with the same growth in the 6-leaf stage were selected and placed in growth trays containing 1/2 Hoagland solution for foam board suspension culture. Daily, the evaporated water was replenished with deionized water, while the nutrient solution was completely updated every three days. The seedlings, nurtured in a growth chamber, experienced a light intensity of 900 μmol m^−2^ s^−1^, a temperature range of 26 ± 2 °C, a photoperiod cycle of 16/8 h (day/night), and a mean relative humidity of 70 ± 10%.

### 4.2. Experimental Design

Se treatment was conducted in the flowering stage of *P. vulgaris* seedlings in May 2023. The uniform seedlings were selected for the acid stress test, with 100 plants placed in growth trays for each treatment. A simulated acid stress solution was created with 1/2 Hoagland nutrient solution, adjusting its pH to 4.0 through a 5:1 molar mix of H_2_SO_4_ and HNO_3_. The half-strength Hoagland nutrient solution without acid solution (pH 6.5) was used as the control. Five treatments were established in the experiment: (1) control +0 mg L^−1^ Se solution (CK); (2) acid stress (pH 4.0) +0 mg L^−1^ Se solution (AS); (3) acid stress (pH 4.0) +1 mg L^−1^ Se solution (AS+S1); (4) acid stress (pH 4.0) +5 mg L^−1^ Se solution (AS+S5); and (5) acid stress (pH 4.0) +10 mg L^−1^ Se solution (AS+S10). Se was dissolved in deionized water, resulting in five prepared concentrations, namely 0, 0, 1, 5, and 10 mg L^−1^, with selenium treatment solutions prepared using sodium selenite (Na_2_SeO_3_, non-hydrated, purity 97%, produced in Tianjin chemical reagent factory, China). At about 6:00 p.m., the foam boards with fixed seedlings were taken out and erected on the ground. Subsequently, the corresponding selenium solutions were sprayed on the leaves of the above different treatments, respectively. The solution on the leaves was dried after spraying, and the Se solution sprayed on the foam board was wiped with a cloth in time to avoid the entry of residual Se solution into the nutrient solution, and then the seedlings were put back into the growth trays. All treatment solutions were sprayed on the leaves for 3 consecutive days. A tri-daily complete replacement of the nutrient solution was conducted, along with daily assessments of pH and evaporation to ensure that the growth of *P. vulgaris* was influenced only by selenium. After a 15-day treatment period, the fluorescence, photosynthetic, and biochemical indices were measured. In late June, the entire plants of *P. vulgaris* were harvested, and their growth, yield characteristics, and medicinal components were subsequently evaluated.

### 4.3. Determination of Antioxidant Enzyme Activity

For the preparation of the homogenate, 0.25 g of fresh leaves were combined with 10 mL of 0.05 mM phosphate-buffered saline (PBS, pH 7.8) solution and 1% PVP in an ice bath. After centrifugation at 3000 rpm for 10 min with H1850R table-top high-speed freezing centrifuge (Hunan Xiangyi Lab Instrument Development Co., Ltd., Changsha, China), the supernatant was employed to measure the enzymatic activities of SOD, POD, and APX. As per the procedure outlined by Giannopolitis and Ries [86], the reaction mixture consisted of 3 mL containing 75 μM nitroblue tetrazolium (NBT), 130 mM methionine, 100 μM ethylenediamine tetraacetic acid, 20 μM riboflavin, and 0.2 mL of the enzyme extract. The absorbance of the samples was determined at 560 nm after illumination for 15 min. SOD activity was defined as the ability to inhibit the reduction of NBT by 50%. The activity of POD was assayed using the previously described method [87]. The reaction mixture included 50 mM PBS (pH 6.0), 100% guaiacol solution, 30% H_2_O_2_ (*v*/*v*), and 0.5 mL of the enzyme extract. POD activity was assessed by measuring absorbance at 470 nm. For APX activity [88], 3 mL of the reaction mixture contained 1.8 mL of PBS (50 mM, pH 7.0), 0.1 mL of ascorbic acid (15 mM), 1 mL of H_2_O_2_ (0.3 mM), and 0.1 mL of the enzyme extract. After the addition of H_2_O_2_, the activity of APX was determined by measuring the change in absorbance at 290 nm. The activity of GSH-px in *P. vulgaris* was determined using a plant GSH-Px kit (Suzhou Grace Biotechnology (China) Co., Ltd., Suzhou, China). Glutathione (GSH) can react with 5,5′-dithiobis (2-nitrobenzoic acid) to generate a yellow substance which has an absorption peak at 412 nm. GSH-px catalyzes the oxidation of glutathione (GSH) by the organic peroxide reagent, reducing the content of GSH. This kit determines the relevant indexes of GSH-px by detecting the absorption of the yellow substance at 412 nm.

### 4.4. Hydrogen Peroxide, Superoxide Anion, MDA Content, and Electrolyte Leakage

The H_2_O_2_ levels were quantified using the Velikova et al. [89] method. Fresh leaves (0.5 g) were homogenized with cooled 1% trichloroacetic acid, centrifuged, and the supernatant mixed with PBS (10 mM, pH 7.0) and KI (1 M). After reacting in darkness for 60 min, the H_2_O_2_ concentration was measured spectrophotometrically at 390 nm using a 752 N instrument.

The superoxide anion (O_2_^−^) levels were assessed using the protocol by Elstner and Heupel [90]. A 0.5 g aliquot of fresh leaves was macerated in 65 mM PBS (pH 7.8) to obtain a homogenate. The assay mixture was prepared with 1 mL homogenate, 1.5 mL of 65 mM PBS (pH 7.8), and 0.5 mL NH_2_OH·HCl (10 mM) and incubated at 25 °C for 15 min. Subsequently, 17 mM p-aminobenzene sulfonic acid and 7 mM alpha-naphthylamine were added for a 30 °C incubation for 30 min. The O_2_^−^ concentration was spectrophotometrically determined at 530 nm.

MDA levels were determined using the method of Heath and Packer [91], beginning with the homogenization of 1.0 g leaves in 10 mL 5% trichloroacetic acid (TCA), followed by centrifugation at 3000 rpm for 20 min with H1850R table-top high-speed freezing centrifuge (Hunan Xiangyi Lab Instrument Development Co., Ltd., China) to obtain the supernatant. A 2 mL aliquot of this supernatant was combined with 2 mL of 0.67% thiobarbituric acid (TBA) solution, which was prepared with 10% TCA. and heated to boiling point for 30 min. The MDA concentration was calculated from the absorbance readings at 600 nm, 532 nm, and 450 nm.

Electrolyte leakage (EL) was evaluated according to Dionisio-Sese and Tobita [92], by slicing 0.2 g of fresh leaf tissue into strips and immersing them in deionized water for 24 h at ambient temperature to achieve equilibrium, after which initial conductivity (C1) was recorded. The samples were then boiled for 30 min to measure the secondary conductivity (C2). EL percentage was calculated as (C1/C2) × 100%.

### 4.5. Determination of Pigment Contents and Gas Exchange Parameters

Following the previously described methodology [93], the measurement of pigment concentrations was performed. Twenty fresh leaves of *P. vulgaris* were randomly selected from each treatment. The surface contaminants were washed off with distilled water, and the surface moisture was dried. After being cut into pieces and mixed together, 0.03 g was randomly weighed out and placed into a test tube with 5 mL of 80% acetone solution, and the tube was sealed it with plastic film. Following this, the samples were kept in darkness for 48 h. The abundances of Chl a, Chl b, and carotenoids (Car) were evaluated after analyzing the absorbance levels at 470 nm, 646 nm, and 663 nm, respectively. For each treatment, four biological replicates were chosen for analysis.

During the early morning hours from 9:00 to 11:00 a.m. on clear days, the photosynthetic indicators, encompassing the net photosynthetic rate (P_n_), stomatal conductance (G_s_), intercellular CO_2_ level (C_i_), and transpiration rate (T_r_), were assessed utilizing the portable photosynthetic apparatus, LI-6400 (manufactured by LI-COR, Lincoln, NE, USA). The leaf chamber conditions were controlled as follows: leaf temperature, 25 °C; airflow rate, 500 μmol s^−1^; CO_2_ concentration, 380 μmol mol^−1^; and photon flux density, 1000 µmol m^−2^ s^−1^. Each treatment employed six biological replicates to ensure reliable and reproducible results.

### 4.6. Determination of Osmolyte Content

The level of proline was determined according to the method of Bates et al. [94], 0.20 g of leaves were dipped in 5 mL of 3% sulfosalicylic acid and heated to 100 °C to isolate the proline. Following this, equal volumes of 2 mL of pure acetic acid and 2 mL of 2.5% solution ninhydrin were mixed with the extraction solution, and the blend was re-boiled for 30 min. Upon cooling, 4 mL of pure toluene was introduced, the mixture was vigorously agitated using a vortex mixer, and then centrifuged at 3000 rpm with H1850R table-top high-speed freezing centrifuge (Hunan Xiangyi Lab Instrument Development Co., Ltd., China). The concentration of proline was estimated by analyzing the absorbance at 520 nm.

The quantification of soluble sugars in leaves was carried out as per the previous methods [95]. Approximately 0.10 g of fresh leaves were sliced into small pieces and extracted twice, with each extraction being a 30 min treatment in boiling water. Afterwards, the extract was added to 0.5 mL of 0.2% anthrone ethyl acetate and 5 mL of 98% concentrated sulfuric acid, followed by boiling for 1 min. Then, the spectrophotometric evaluations at 630 nm were performed to calculate the soluble sugar content.

Using previous methods [96], the soluble protein content was determined. Eight milliliters of deionized water containing 1% PVP was added to 0.20 g of leaf tissue for homogenization, followed by centrifugation at 1000 rpm with H1850R table-top high-speed freezing centrifuge (Hunan Xiangyi Lab Instrument Development Co., Ltd., China). Subsequently, 2.5 mL of Coomassie Brilliant Blue G-250 was combined with 0.5 mL of the supernatant obtained and allowed to react for 2 min. Lastly, the soluble protein content was quantified by assessing the absorbance at a wavelength of 595 nm.

### 4.7. Root Architecture Analysis

The excised roots were thoroughly rinsed with distilled water. Subsequently, a scanner (Epson Expression 11000XL, Seiko Epson Corporation, Yokohama, Japan) was utilized to capture detailed images of the roots (Figure S1). For each treatment, the root architecture of 12 plants was analyzed using the root analysis system (WinRHIZO, Regent Instruments, Inc., Quebec City, Canada).

### 4.8. Determination of Morphological and Yield Traits

The quantification of branches and spicas per plant was conducted through conventional practices. The exact dimensions of length and width for each spica were ascertained with a vernier caliper. Subsequently, the whole plants of *P. vulgaris* were dried to a stable quality. Following this, the weights of both the dried spica and the entire plant were precisely determined utilizing an electronic balance (FA1204B; Jingke Tianmei Scientific Technological Instrument, Shanghai, China). For each treatment, twelve seedlings were randomly selected to ensure representative sampling.

### 4.9. Fast Chlorophyll Fluorescence Induction Kinetic Curve (OJIP) and 820 nm Modulated Reflection

The OJIP transients and the modulated reflected beam (MR) at 820 nm, as reported by Strasser et al. [81], were studied using the M-PEA instrument (Hansatech, Norfolk, UK). OJIP transient was obtained by irradiating the third or fourth inverted leaf of Prunella vulgaris with red light intensity of 3000 μmol m^−2^ s^−1^ after complete dark adaptation for 30 min (Table 4) [81]. The calculation of V_t_, representing the normalized relative variable fluorescence at any time, was performed, accompanied by the quantification of the difference in Vt (ΔV_t_) existing between the selenium-treated and control sets [97]: V_t_ = (F_t_ − F_0_)/(F_m_ − F_0_) and ΔV_t_ = V_t(Se)_ − V_t(CK)_. The modulated reflection was measured at 820 nm under simultaneous exposure to 250 μmol m^−2^ s^−1^ far-red light. Based on this modulation reflection curve, the maximal redox activity of PSI (ΔI/I_0_) and the coordination of PSII and PSI (Φ_(PSII/PSII)_) were determined by applying the corresponding formulas: ΔI/I_0_ = (I_0_ − I_m_)/I_m_ and Φ_(PSII/PSII)_ = (ΔI/I_0_)/ψ_0_, respectively [98,99]. Each treatment was subjected to at least 10 biological replicates.

### 4.10. Determination of Secondary Metabolites Content

The content of total flavonoids in spicas was measured by previously described methods [10]. After being mixed with 10 mL of 35% ethanol solution, 0.2 g of dried spica powder was extracted in an 86 °C water bath for three times. Then, the extract was reacted with 0.3 mL of 5% NaNO_2_ for 6 min, after which 0.3 mL of 10% Al(NO_3_)_3_ was added and the mixture was left to stand for another 6 min. Finally, 4 mL of 4% NaOH was added to react for 15 min, and the absorbance of the solution was determined at 510 nm (752 N, INESA, Shanghai, China). The addition of 0.25 g of dried *P. vulgaris* spica powder to 25 mL of a blend of 80% methanol and 1% formic acid led to its ultrasonic extraction at 50 °C for 30 min. Following this, the resulting supernatant underwent filtration through a 0.22 μm organic membrane filter and was stored in a 1.5 mL brown bottle. The extracts were quantitatively analyzed using an Agilent 1260 High-Performance Liquid Chromatography (HPLC) instrument (Agilent Technologies, Inc., Santa Clara, CA, USA) with a Waters C18 column (250 mm × 4.6 mm).

The previous method [10] was followed to establish the standard curves for chromatographically pure caffeic acid (CAS 331-39-5), ferulic acid (CAS 1135-24-6), rosmarinic acid (CAS 20283-92-5), and hyperoside (CAS 482-36-0), all of which were procured from Shanghai Yuanye Biotech Co., Ltd., (Shanghai, China). The concentrations of the four medicinal components, having been measured at 325 nm, were subjected to calculations using their respective reference substances. The resulting values were expressed as mg g^−1^ (dry weight).

### 4.11. qRT-PCR Analysis

The mRNA levels of *C4H*, *4CL*, *PAL*, and *TAT* was quantified by real-time quantitative fluorescence PCR (qRT-PCR). The total RNA was extracted from leaves using the RNAiso Plus Total RNA Extraction Kit (Beijing Genomic Biotechnology Co., Ltd., Beijing, China). The cDNA was produced using the M5 Super-qPCR RT Kit (Mei5 Biotechnology Co. Ltd., Beijing, China), as described in the accompanying manual. Primer Premier 5.0 (Oakville, ON, Canada) was used to design 4CL primers from the target sequence (Table 5), and other primers were obtained from a previously published reference [100]. The CFX96 Real-Time PCR System (Bio-rad, Hercules, CA, USA) was utilized to perform the RT-PCR reaction, adhering to the PCR protocol as described previously [100]. Utilizing the 2^−ΔΔCt^ method, the relative expression of genes was computed, with normalization performed against the transcription level of β-actin in *P. vulgaris*, with three biological repetitions.

### 4.12. Statistical Analysis

The means were compared for significant differences using the Duncan test available in SPSS (version 13, New York, IL, USA). The physio-biochemical parameters of all treatments were determined using a minimum of three biological replicates. The results were presented in the form of means ± standard deviations.

## 5. Conclusions

Our findings revealed that acid stress stimulated an enhancement in enzymatic antioxidant activity and nonenzymatic substances in *P. vulgaris*. However, acid stress significantly hindered the growth of roots and shoots, disrupted membrane integrity, decreased photosynthesis, and ultimately led to a marked decrease in the yield of *P. vulgaris*. Conversely, the application of Se exerted a profound amplifying effect on the enzymatic antioxidant system and nonenzymatic active substances in *P. vulgaris* subjected to acid stress. It efficiently preserved the integrity of the membrane system, significantly promoted photosynthetic performance, and stimulated the growth of the root system and above-ground parts, thereby increasing the yield of *P. vulgaris*. Additionally, Se application noticeably augmented the medicinal components and key gene expression of *P. vulgaris*. Our findings indicated that Se conferred greater acid resistance to *P. vulgaris* by strengthening its antioxidant defense mechanisms, photosynthetic capabilities, growth and key gene expression. Of the selenium treatments assessed, the 5 mg L^−1^ selenium dosage provided the most marked improvement in the growth, yield, and quality of *P. vulgaris* under acidic stress. *P. vulgaris*, recognized for its extensive medicinal uses, can benefit from foliar selenium application to enhance its growth and medicinal compound content under acidic stress, which would positively impact the cultivation of *P. vulgaris* in the southern regions of China, particularly in areas where acidic stress is prevalent.

## Figures and Tables

**Table 1:** Changes in root architecture of *P. vulgaris* under different Se treatments.

Se Treatment	Root Length (cm)	Root Surface Area (cm^2^)	Root Volume (cm^3^)	The Number of Root Tip	Branch Number
CK	760 ± 70 a	112.41 ± 13.11 a	1.41 ± 0.15 a	1409 ± 137 b	3133 ± 271 a
AS	342 ± 34 e	43.14 ± 4.42 d	0.44 ± 0.08 c	593 ± 135 d	1159 ± 230 c
AS+S1	595 ± 23 c	73.59 ± 5.58 bc	0.73 ± 0.10 b	1128 ± 105 c	2525 ± 415 b
AS+S5	695 ± 49 b	80.81 ± 10.29 b	0.77 ± 0.15 b	1743 ± 126 a	2840 ± 443 ab
AS+S10	529 ± 30 d	65.16 ± 6.95 c	0.64 ± 0.12 b	1143 ± 161 c	2358 ± 226 c

CK: control; AS: acid stress: AS+S1, AS+S5, and AS+S10: treated with acid stress and 1, 5, and 10 mg L^−1^ Se solution, respectively. After treatment with the selenium spray, the seedlings underwent 15 days of acid stress and were subsequently tested. The data are represented as the mean ± standard deviation. Significant differences are indicated by different lowercase letters above the bars according to a Duncan’s multiple range test (*p* < 0.05).

**Table 2:** The changes in energy fluxes in *P. vulgaris* leaves under different Se treatments.

Parameter	CK	AS	AS+S1	AS+S5	AS+S10
ABS/RC	1.43 ± 0.06 c	1.72 ± 0.05 a	1.58 ± 0.13 b	1.56 ± 0.04 bc	1.62 ± 0.05 ab
DI_0_/RC	0.31 ± 0.03 c	0.41 ± 0.02 a	0.34 ± 0.02 bc	0.32 ± 0.03 bc	0.37 ± 0.04 ab
Tr_0_/RC	1.12 ± 0.04 c	1.31 ± 0.03 a	1.24 ± 0.05 b	1.22 ± 0.01 b	1.25 ± 0.03 b
ET_0_/RC	0.65 ± 0.03 a	0.64 ± 0.09 a	0.65 ± 0.07 a	0.70 ± 0.02 a	0.64 ± 0.10 a
φD_0_	0.19 ± 0.01 c	0.26 ± 0.00 a	0.23 ± 0.01 b	0.21 ± 0.01 bc	0.23 ± 0.02 b
ABS/CS_m_	31,141.33 ± 845.22 a	28,070.67 ± 714.81 b	29,996.67 ± 1295.91 a	30,211.00 ± 438.63 a	30,518.00 ± 1253.21 a
DI_0_/CS_m_	6332.00 ± 346.17 a	6607.67 ± 527.45 a	6734.00 ± 932.52 a	6251.00 ± 685.96 a	7011.00 ± 577.05 a
Tr_0_/CS_m_	24,809.33 ± 569.70 a	21,463.00 ± 730.87 b	23,262.67 ± 2086.98 ab	23,960.00 ± 789.86 a	23,507.00 ± 1304.77 ab
ET_0_/CS_m_	14,121.00 ± 531.10 a	10,493.67 ± 784.19 b	12,201.33 ± 1351.51 ab	13,520.33 ± 251.29 a	12,132.33 ± 1697.49 ab
RC/CS_m_	18,159.41 ± 1290.96 a	13,420.75 ± 655.54 d	1556.20 ±231.12 bc	16,415.79 ± 870.11 b	14,845.98 ± 415.26 cd

CK: control. AS: acid stress. AS+S1, AS+S5, and AS+S10: treated with acid stress and 1, 5, and 10 mg L^−1^ Se solution, respectively. After treatment with the selenium spray, the seedlings underwent 15 days of acid stress and were subsequently tested. The data are represented as the mean ± standard deviation. Significant differences are indicated by different lowercase letters above the bars according to a Duncan’s multiple range test (*p* < 0.05).

**Table 3:** Effects of exogenous selenium on the contents of total flavonoids, caffeic acid, ferulic acid, rosmarinic acid, and hyperoside in *P. vulgaris* (mg g^−1^_DW_).

Se Treatment	Total Flavonoids	Caffeic Acid	Ferulic Acid	Rosmarinic Acid	Hyperoside
CK	58.56 ± 2.22 d	0.09 ± 0.00 b	0.55 ± 0.01 d	6.06 ± 0.04 c	0.34 ± 0.01 c
AS	62.89 ± 2.38 c	0.09 ± 0.00 b	0.58 ± 0.01 c	6.43 ± 0.18 b	0.36 ± 0.01 c
AS+S1	67.98 ± 2.05 b	0.10 ± 0.01 b	0.76 ± 0.01 b	6.69 ± 0.19 b	0.40 ± 0.02 b
AS+S5	82.40 ± 0.72 a	0.11 ± 0.01 a	0.82 ± 0.02 a	7.40 ± 0.16 a	0.44 ± 0.02 a
AS+S10	80.84 ± 0.88 a	0.12 ± 0.00 a	0.66 ± 0.01 c	7.51 ± 0.16 a	0.41 ± 0.03 ab

CK: control. AS: acid stress. AS+S1, AS+S5, and AS+S10: treated with acid stress and 1, 5, and 10 mg L^−1^ Se solution, respectively. After treatment with the selenium spray, the seedlings underwent 15 days of acid stress and were subsequently tested. The data are represented as the mean ± standard deviation. Significant differences are indicated by different lowercase letters above the bars according to a Duncan’s multiple range test (*p* < 0.05).

**Table 4:** The JIP parameters.

Fluorescence Parameters	Description
W_K_ = (F_K_ − F_0_)/(F_J_ − F_0_)	Normalized relative variable fluorescence
V_J_ = (F_J_ − F_0_)/(F_m_ − F_0_)	Relative variable fluorescence intensity at the J step
M_0_ = 4 (F_300μs_ − F_0_)/(F_m_ − F_0_)	The initial slope of the relative variable fluorescence of the relative rate at which *Q*_A_ is reduced
φE_0_ = ET_0_/ABS = [1− (F_0_/F_m_)]ψ_0_	Quantum yield for electron transport
ABS/RC = M_0_ (1/V_J_) (1/φP_0_)	Absorption flux per reaction center
TR_0_/RC = M_0_(1/V_J_)	Trapped energy flux per reaction center (RC)
ET_0_/RC = M_0_ (1/V_J_) ψE_0_	Electron transport flux per RC
DI_0_/RC = (ABS/RC) − (TR_0_/RC)	Dissipated energy flux per RC
RC/CS_m_ = φP_0_ (V_J_/M_0_) (ABS/CS_m_)	Density of RCs per excited cross section (CS)
ABS/CS_m_	Absorbed energy flux per CS
TR_0_/CS_m_ = φP_0_(ABS/CS_m_)	Trapped energy flux per CS
ET_0_/CS_m_ = φE_0_(ABS/CS_m_)	Electron transport flux per CS
DI_0_/CS_m_ = ABS/CS_m_-TR_0_/CS_m_	Dissipated energy flux per CS
F_v_/F_m_	The maximal quantum yield of PSII photochemistry
φD_0_	Quantum yield of energy dissipation
PI_ABS_ = (RC/ABS) [φP_0_/(1 − φP_0_)][ψ_0_/(1 − ψ_0_)]	Performance index on absorption basis

**Table 5:** Primers used for qRT-PCR analysis.

Gene	Genbank Accession Number	Primer Name	Primer Sequence (5′ → 3′)	PCR Product (bp)
*PvC4H*	KJ010816	*PvC4H* forward	ATCGTTGTCGCCGCCGTTGTGT	136
		*PvC4H* reverse	CGTAGTCGGTGAGGTTTCGGTGGTTC	
*Pv4CL*	KJ010817.1	*Pv4CL* forward	CCACCATGGCCAATCCCTATT	114
		*Pv4CL* reverse	CATAGTCCCGCACCTTGTCG	
*PvPAL*	KJ010815.1	*PvPAL* forward	TCCGTGCTTGTGTGTTTGTGCCTGTC	*203*
		*PvPAL* reverse	GGCTTCCTGAACTCCTCCACCATCCT	
*PvTAT*	KM053278	*PvTAT* forward	CGTCTACTCGCATCAGCATCTCAGGA	194
		*PvTAT* reverse	GCCAACCAGGGATCAACCACCTCTTC	
β-actin	KJ010818	β-actin forward	GCAGTTCTCTCCCTATACGCCAGTGG	205
		β-actin reverse	GCTCGGCTGTGGTGGTGAATGAGTAA	

## Data Availability

The original contributions presented in the study are included in the article.

## Supplementary Materials

The following supporting information can be downloaded at: https://www.mdpi.com/article/10.3390/plants14060920/s1, Figure S1. Effects of exogenous selenium on roots in *P. vulgaris* under acid stress. CK: control. AS: acid stress. AS+S1, AS+S5, and AS+S10: treated with acid stress and 1, 5, and 10 mg L−1 Se solution, respectively. After treatment with the selenium spray, the seedlings underwent 15 days of acid stress and were subsequently tested.
