# Supplementary material for: Selenium Improves Yield and Quality in Prunella vulgaris by Regulating Antioxidant Defense, Photosynthesis, Growth, Secondary Metabolites, and Gene Expression Under Acid Stress
Source: Plants (Basel). 2025 Mar 14;14(6):920. doi: 10.3390/plants14060920 (PMC11944784; doi:10.3390/plants14060920)
Supplement: Supplementary file 1 [file plants-14-00920-s001.zip › plants-3492815-supplementary.pdf]

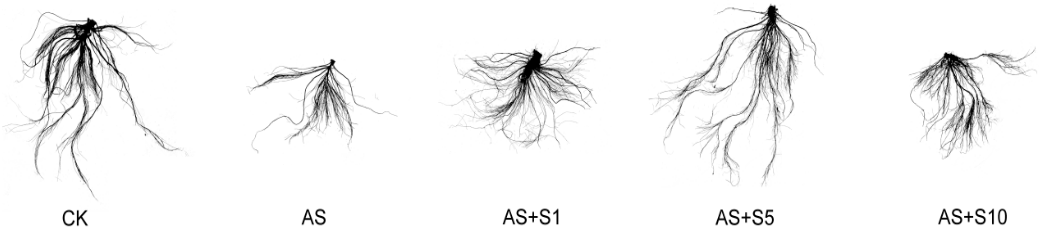

Figure S1. Effects of exogenous selenium on roots in *P. vulgaris* under acid stress. CK: control. AS: acid stress. AS+S1, AS+S5, and AS+S10: treated with acid stress and 1, 5, and 10 mg L−1 Se solution, respectively. After treatment with the selenium spray, the seedlings underwent 15 days of acid stress and were subsequently tested
